# Serum Amino Acids Imbalance in Canine Chronic Hepatitis: Results in 16 Dogs

**DOI:** 10.3390/vetsci9090455

**Published:** 2022-08-25

**Authors:** Verena Habermaass, Eleonora Gori, Francesca Abramo, Francesco Bartoli, Alessio Pierini, Chiara Mariti, Ilaria Lippi, Veronica Marchetti

**Affiliations:** 1 Department of Veterinary Sciences, Veterinary Teaching Hospital “Mario Modenato”, University of Pisa, Via Livornese Lato Monte, San Piero a Grado, 56122 Pisa, Italy; 2 Department of Translational Research and New Technologies in Medicine and Surgery, University of Pisa, Via Savi 10, 56126 Pisa, Italy

**Keywords:** canine chronic hepatitis, dog hepatopathies, canine liver disease, serum amino acids, BCAAs, BCAAs/AAAs ratio, fibrosis score, liver histology, liver disease metabolomic, High Performance Liquid Chromatography

## Abstract

**Simple Summary:**

Human chronic liver disease is reported to be associated with alterations in amino acids metabolism, with a decrease in serum branched-chain amino acids and an increase in aromatic amino acids. A decreased Fischer ratio (branched to aromatic amino acids ratio) has showed prognostic significance and is a therapeutic target in human cirrhosis. In dogs, few studies have been performed, and the Fischer ratio seems to be reduced in animals with congenital portosystemic shunts. The aim of this study was to evaluate serum amino acids in dogs with chronic hepatic inflammation compared with healthy dogs. The serum amino acids in dogs with chronic hepatitis were also evaluated in relation to their histological severity. Eighteen amino acidic metabolites were measured using the leftover serum samples of 16 dogs with histological chronic hepatitis and 25 healthy dogs. Several amino acid concentrations were significantly different between dogs diagnosed with chronic hepatitis and healthy controls. In human medicine, aromatic amino acids seem to increase during chronic hepatitis, whereas isoleucine decreases. The Fischer ratio was significantly reduced if higher grades of fibrosis were present. Even if total serum proteins did not significantly differ between groups, we observed qualitative imbalances in serum amino acids among dogs presenting with chronic hepatitis.

**Abstract:**

In humans, chronic liver disease may cause alterations in amino acids (AAs) metabolism, with serum branched-chain AAs (BCAAs) decreasing and aromatic AAs (AAAs) increasing. A reduced Fischer ratio (BCAAs/AAAs) has been found to be associated with hepatic fibrosis and is useful for assessing prognosis in human patients. In veterinary medicine, few studies have been performed, and in contrast to human patients, dogs with different kinds of hepatopathy tend to show both increased AAAs and BCAAs. In dogs, the association between histological scores and serum AAs has not been previously investigated. The aim of this study was to evaluate serum AAs in dogs with chronic hepatitis (CH) compared with a healthy control group (C) and, among CH dogs, in relation to their histological fibrosis and necroinflammatory activity scores. Leftover serum samples of 16 dogs with histological CH and 25 healthy dogs were employed. Serum AAs were measured by high performance liquid chromatography. Proline and the AAAs phenylalaine and tyrosine progressively increased with the histological severity. In contrast, cysteine, tryptophan and BCAA isoleucine progressively reduced. Lysine and the BCAAs leucine and valine showed a non-linear trend with the histological findings. The BCAAs/AAAs ratio was significatively reduced if higher grades of liver fibrosis were present.

## 1. Introduction

The liver plays a central role in the metabolism of proteins, carbohydrates, lipids, fatty acids and amino acids involved in both anabolic and catabolic processes, and chronic hepatopathies results in important metabolic imbalances. Regardless of the cause, both primary and secondary chronic liver diseases can result in metabolic changes that can affect quality of life and life expectancy [1,2,3,4]. It has been recognized that during human cirrhosis, serum concentrations of branched-chain amino acids (BCAAs), such as leucine, isoleucine, and valine, are decreased, whereas those of aromatic amino acids (AAAs), such as phenylalanine and tyrosine, are increased [5]. Decreased Fischer ratios (BCAAs/AAAs) and BTR (BCAAs/tyrosine) ratios are reported to be useful parameters to assess the prognosis in cirrhotic human patients [6,7,8]. Moreover, the serum concentration of tyrosine has been reported to increase early during chronic liver disease in humans and showed a positive correlation with histologic fibrosis scores [9]. In human medicine, the administration of BCAAs has shown positive results in patients, preventing progressive hepatic failure and improving survival [10,11,12,13,14,15].

The above-mentioned effects of cirrhosis are not well characterized in veterinary medicine. The interest in the past decades has been mainly focused on serum amino acids and aminoaciduria in dogs with hepatocutaneous syndrome and generalized hypoaminoacidemia [16]. The few studies present in veterinary medicine are mainly focused on AAs alterations in congenital portosystemic shunts (PSS). These studies show that the trend of the BCAAs and BCAAs/AAAs ratios was reduced only in PSS as an expression of liver failure [17]. Similarly, increased BCAAs were found in dogs with uncharacterized elevated liver enzymes and decreased BCAAs were found in PSS dogs, and for both groups, increased phenylalanine and tyrosine were observed [18]. Therefore, in contrast to human patients, dogs with different kinds of hepatopathy tend to show increased BCAAs [17,18,19,20,21].

The aim of this study was to evaluate serum AAs pattern in dogs with histologically confirmed chronic hepatitis (CH) compared with a healthy control group. Our aim was also to evaluate AAs patterns among CH dogs, with a focus on differences in AAs in relation to dogs’ histological fibrosis and necroinflammatory scores.

## 2. Materials and Methods

### 2.1. Study Population and Sample Preparation

This prospective case-control study on client-owned dogs with a histological diagnosis of chronic hepatitis was conducted at the internal medicine service of the Veterinary Teaching Hospital “Mario Modenato” of the University of Pisa, between January 2020 and January 2022 (Ethic committee approval 41/2020).

The diagnosis of chronic hepatitis was based on history, physical examination, hematology and biochemistry, abdominal ultrasonography, and histologic features according to WSAVA guidelines (Standards for Clinical and Histological Diagnosis of Canine and Feline Liver Disease) [22]. Routinely, hepatic biopsies were performed through laparotomy or laparoscopy, and formalin-fixed paraffin-embedded histology samples were examined by a pathologist with expertise in histological liver diseases. For each CH dog, necroinflammatory activity (A) and fibrosis (F) scores were assessed according to current WSAVA guidelines [22]. Necroinflammatory activity was graded as A0 = absent, A1 = slight, A2 = mild, A3 = moderate, A4 = marked, or A5 = very marked [22]. The dogs were then divided into necroinflammatory activity groups: A0–2 (absent to mild) and A3–5 (moderate to very marked). Histological fibrosis was also graded as 0 = absent, 1 = mild, 2 = moderate, 3 = marked, or 4 = very marked, and dogs were divided into F0–2 and F3–4 groups according to their fibrosis score [23,24,25].

A control group of healthy blood-donor dogs was also included. Prior to each blood donation, blood-donor dogs routinely underwent a clinical evaluation and complete blood work (hematological and biochemical evaluation and a serology test for Leishmaniosis and other tick-borne diseases).

Dogs with AAs supplementation in their recent clinical history, dogs presenting proteinuria (urinary protein-to-creatinine ratio > 2) with inactive urinary sediment, and dogs with suspected protein losing enteropathy, were excluded. Information about diet, Body Condition Score (BCS), Muscle Condition Score (MCS), total serum proteins, albumin, alkaline phosphatase (ALP), alanine aminotransferase (ALT), aspartate aminotransferase (AST), gamma-glutamyl transferase (GGT), and medical therapy according to the latest consensus statement [26] (hepatoprotectors, corticosteroids and/or cyclosporin) at the time of inclusion were recorded.

Blood samples were taken from the jugular vein for each dog after a 12 h fasting period. Blood samples for hematological analysis were collected in ethylene diamine tetra-acetic acid (EDTA)-coated tubes, whereas those for biochemical analysis were collected in serum-separating tubes. Within a maximum of 15 min from blood collection, the samples were centrifugated and submitted for the routine analysis, including ALP, ALT, AST, GGT, total serum protein (Liasys, Assel SRL, Rome, Italy). Leftover serum quotes were placed in Eppendorf-tubes and frozen at −18 °C within 24 h, and subsequently stored at −80 °C. Employed samples were not preserved for more than 24 months. For both groups, the serum samples used for determination of amino acids were derived from excess serum aliquots, which are routinely stored (−80 °C) for research purposes.

Serum AAs, including Glycine (GLY), L-Alanine (ALA), L-Valine (VAL), L-Leucine (LEU), L-Isoleucine (ILE), Proline (PRO), L-Serine (SER), L-Threonine (THR), L-Cysteine (CYS), L-Methionine (MET), L-Phenylalanine (PHE), L-Tyrosine (TYR), Tryptophan (TRP), L-aspartic acid (ASP), L-glutamic acid (GLU), L-Histidine (HIS), L-Lysine (LYS) and L-Arginine (ARG) were measured by automated high-performance liquid chromatography (HPLC). The samples were prepared using an amino acid analyzer kit (AAA AccQ-Tag, Waters S.p.A.) [27,28]. For each dog, the BCAAs/AAAs ratio was calculated.

### 2.2. Statistical Analysis

Statistical analysis was performed using statistics software SPSS Statistics (IBM Corp., New York, NY, USA). The Kolmogorov–Smirnov test was applied to test for normal distribution of the data. Normally distributed data are expressed as mean ± standard deviation. Non-normally distributed data are expressed as a median and range. An unpaired *t*-test for normally distributed data or a Mann–Whitney U-test for non-normally distributed data were used to investigate differences between the CH and C groups (AAs, BSAAs/AAAs ratio, Total Serum Protein). A Kruskal–Wallis test with Bonferroni correction was employed to evaluate differences in serum AAs among healthy controls and CH dogs considering their A and F scores. The results were considered statistically significant for *p* values < 0.05.

## 3. Results

### 3.1. Animals

The CH group was composed of 16 dogs, 7 females (44%), and 9 males (56%). The median age was 9.5 years (range 2–14). The majority of dogs (*n* = 7; 44%) was mix-breed, followed by Galgo (*n* = 2; 13%) and one each (6%) of the following breeds: Golden Retriever, West Highland White Terrier, Cavalier King Charles Spaniel, French Bulldog, Pincher, Flat Coated Retriever, Pitbull. Regarding BCS, the majority of patients were presenting BCS 4/9 (*n* = 6; 37%) and BCS 5/9 (*n* = 5; 31%), whereas BCS 2/9 (*n* = 1; 6.3%), BCS 6/9 (*n* = 2; 13%), BCS 7/9 (*n* = 1; 6.3%) and BCS 8/9 (*n* = 1; 6.3%) were less represented. Regarding MCS, 5 dogs had an MCS of 2/3 (31%) and 11 dogs (69%) 3/3.

The dogs’ diets were as follows: 2 dogs were fed with home-cooked meals, and 14 with various types of commercial veterinary dry/canned food, both maintenance and gastrointestinal. The median protein content of the diet was 20% (range 13–32%). The median serum protein was 6.15 g/dL (4.4–8.5). The serum biochemical findings of group CH are reported in Appendix A.

The control group was composed of 25 dogs, which consisted of 13 females (54%), and 12 males (46%). The median age was 5.5 years (0.7–12), which was significantly different to the CH group (*p* value = 0.02). Most dogs (*n* = 10; 40%) were mix-breed dogs, followed by Labrador Retriever (*n* = 3; 12%), Golden Retriever (*n* = 3; 12%), two of each of the following breeds (8%): English Setter, Maremma Shepherd, Border Collie, Bernese Mountain Dog, and one Lagotto Romagnolo (*n* = 1; 4%). The mean BCS and MCS were 6 ± 0.6, and 3, respectively. The median serum protein was 6.5 g/dL (5.5–8.1 g/dL). There were no significant differences in serum proteins between the CH group and healthy dogs (*p* = 0.2).

### 3.2. Serum Amino Acids

Several serum AAs had significantly different results in CH dogs compared with healthy controls (Table 1).

### 3.3. Histological Scores

The median histologic A and F scores were 2 (range 1–5) and 2 (range 0–4), respectively. The histological scores A and F and the respective number of assigned dogs are reported in Table 2.

**Figure 1:**
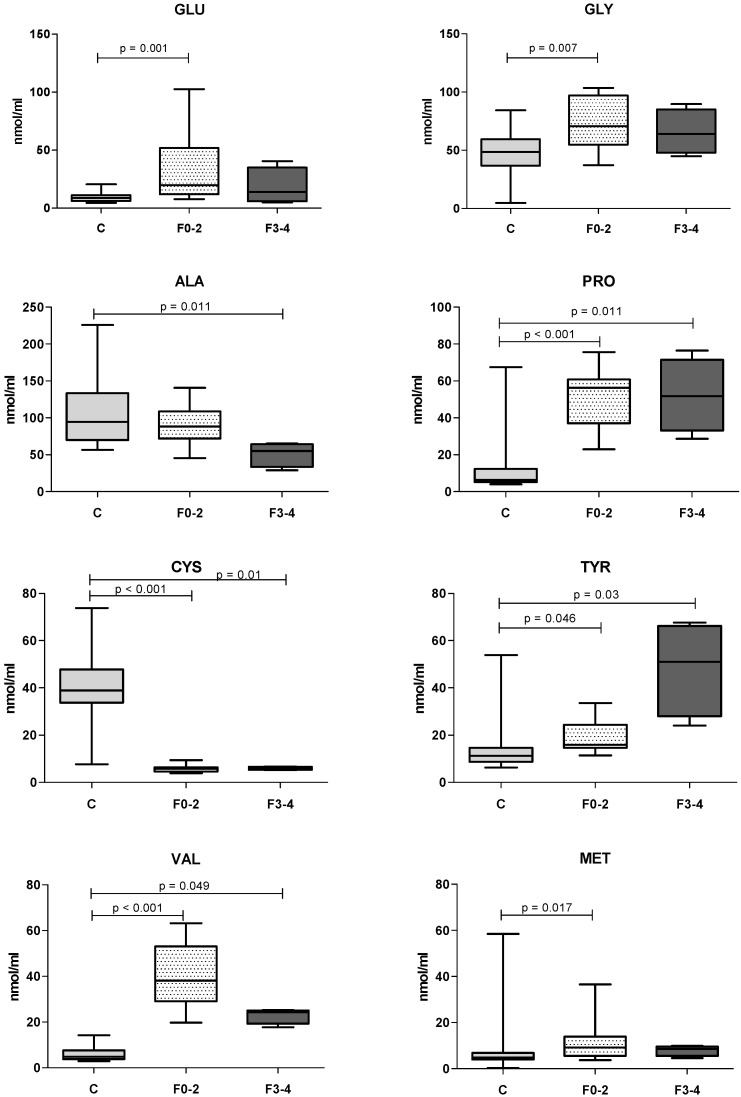

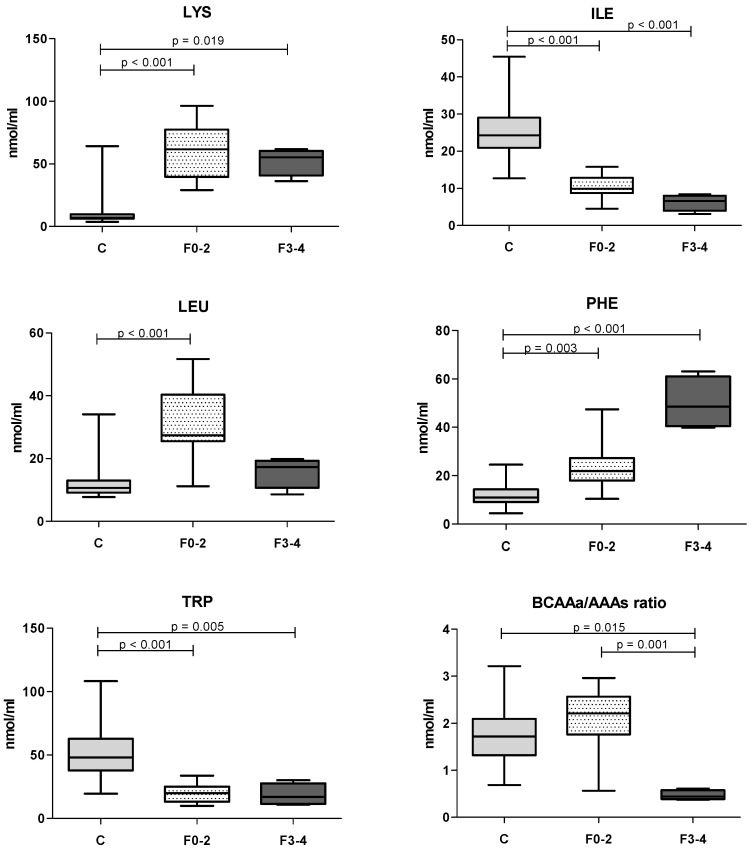
Serum AAs concentrations in healthy controls (C), dogs with lower (F0–2) and higher (F3–4) histological fibrosis scores. Reported AAs did significantly differ after a Kruskal–Wallis test with Bonferroni correction.

We observed differences in AAs levels among dogs presenting marked to very marked fibrosis (F3–4, *n* = 4), absent to moderate fibrosis (F0–2, *n* = 12) and healthy controls (*n* = 25). The trends in AAs in relation to fibrosis scores are shown in Figure 1 below. 

The only significant difference between F0–2 and F3–5 scoring groups was in the BCAAs/AAAs ratio. However, we observed different kinds of trends. GLU, GLY, PRO, VAL, LYS, LEU showed an increase in the F0–2 scoring group and a subsequent reduction in the F3–4 scoring group; AAAs TYR and PHE showed a progressive increase in relation to fibrosis severity. In contrast, ALA and ILE, showed a progressive decrease as the fibrosis grade increased. Finally, in both fibrosis groups MET increased, whereas TRP and CYS decreased.

**Figure 2:**
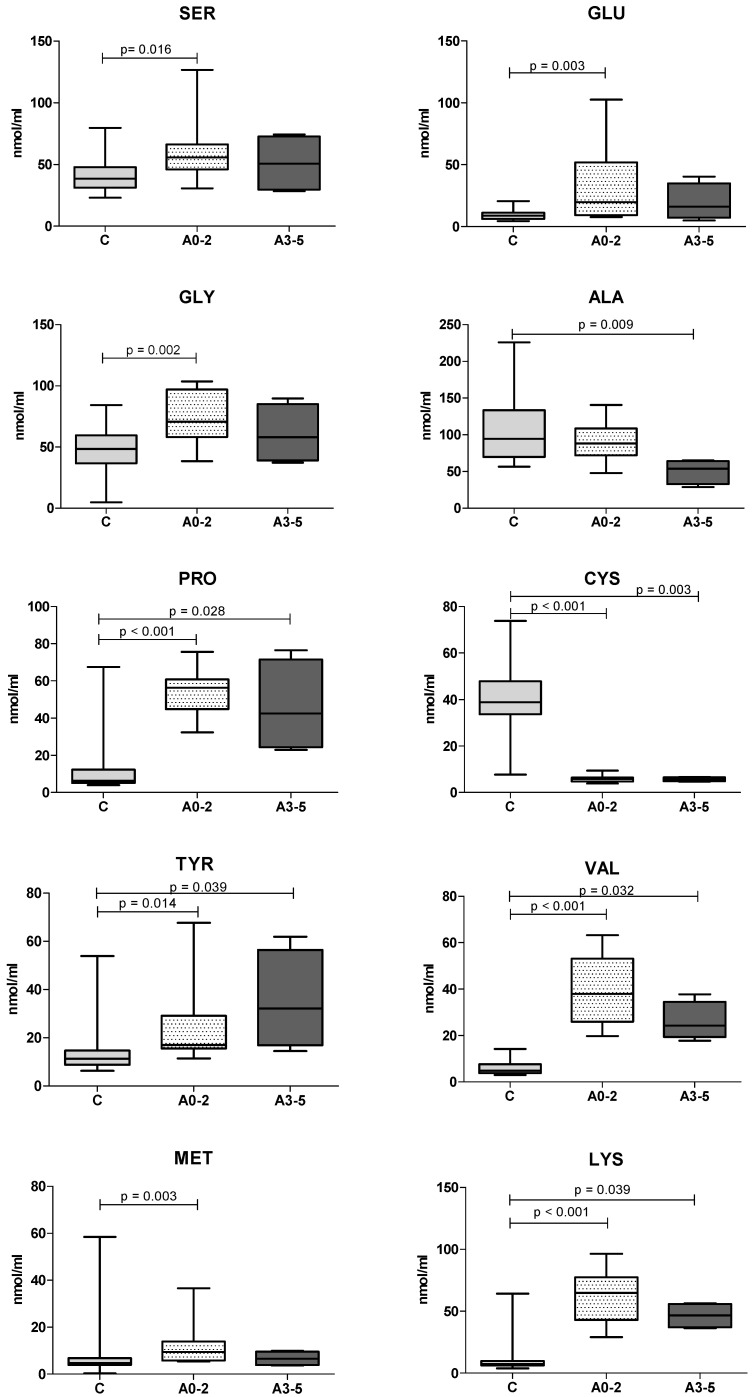

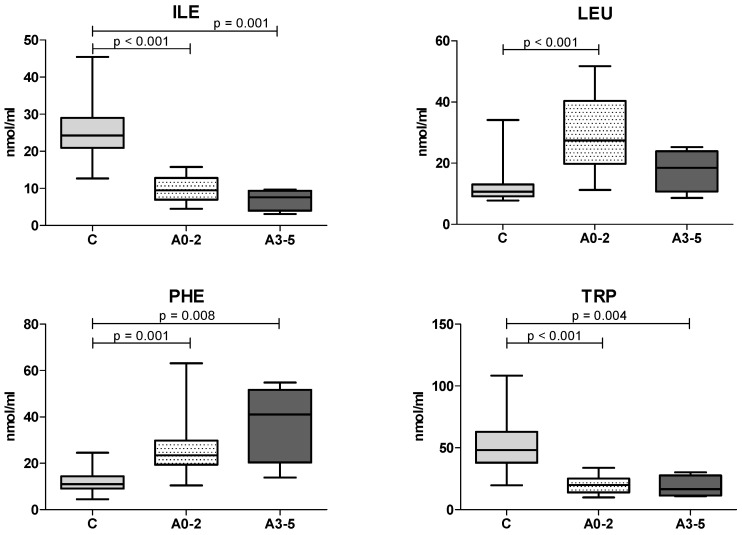
Serum AAs concentration in healthy controls (C), dogs with lower (A0–2) and higher (A3–5) histological necroinflammatory activity scores. Reported AAs did significantly differ after Kruskal–Wallis test with Bonferroni correction.

Regarding the necroinflammatory activity scores, neither the BCAAs/AAAs ratio or measured AAs significantly differed between the A0–2 and A3–5 scoring groups. However, we observed different trends among healthy dogs (*n* = 25), A0–2 scoring group (*n* = 12) and A3–5 scoring group (*n* = 4). The trends in AAs in relation to necroinflammatory activity scores are shown in Figure 2 below. 

Similar to the fibrosis scores, we observed different trends among A3–5, A0–2 scoring groups and healthy controls. GLY, PRO, VAL, LYS, LEU showed an increase in the A0–2 scoring group and a reduction in the A3–5 scoring group, whereas AAAs TYR and PHE showed a progressive increase in relation to inflammatory scores. ALA and ILE showed a progressive decrease as the necroinflammatory grade increased. Lastly, MET, SER, GLU increased in both inflammation groups and TRP and CYS decreased in both necroinflammatory groups.

## 4. Discussion

In this study, several serum AAs concentrations were significantly different between CH and healthy dogs, revealing a potential metabolic amino acidic disorder in CH dogs. The BCAAs/AAAs ratio was decreased in CH dogs, which presented higher grades of hepatic fibrosis, as already reported in human patients.

Our results showed increased concentrations of AAA PHE, similar to the pattern observed in human medicine, and in a previous canine study in which increased serum PHE was considered as a marker of liver disease in dogs [29]. We also observed increased AAA TYR, and decreased BCAA ILE in hepatopathic patients, consistent with a recent study in which six dogs with chronic hepatitis were enrolled [17]. Increased AAAs probably reflect an impaired liver metabolism, as both PHE and TYR are mainly catabolized in the liver [30,31]. Furthermore, PHE and TYR showed a progressive increase, as both fibrosis and necroinflammatory activity scores rose.

CYS showed a progressive decrease as both fibrosis and necroinflammatory activity scores rose. Decreased CYS might be an expression of impaired MET conversion to S-adenosylmethionine by the liver [17]. In contrast, PRO showed a progressive increase as fibrosis scores rose. Increased PRO has never been reported before in canine chronic hepatitis. This finding might reflect an active role of the liver in PRO catabolism [32]. It is also possible that increased LYS was due to the same mechanism, as the liver appears to be the primary site of LYS catabolism [33]. In our population, LYS seemed to be higher in CH dogs, although it showed a non-linear trend in relation to both necroinflammatory activity and fibrosis scores.

TRP was significatively lower in CH dogs, with a progressive reduction in relation to histological severity scores. In previous studies, a reduction in TRP was only reported in different kinds of canine enteropathies [27,34,35].

BCAA ILE showed a progressive decrease as both histopathological scores increased. In contrast to human medicine and similar to a recent study in dogs, other BCAAs (LEU and VAL), were not found to decrease in canine chronic hepatopathies. In human medicine, and to some extent in dogs, one of the most important causes of decreased BCAAs seems to be the hyperammoniaemia related to end-stage liver disease [17,36]. Unfortunately, in the present study, ammonia was not evaluated.

Both LEU and VAL showed a non-linear trend when histological scores were considered, with an apparent increase in lower histological severity grades, followed by a reduction as the severity increased. BCAAs/AAAs did not significantly differ between the CH group and C group, probably as a consequence of increased BCAAs LEU and VAL.

Since the liver provides many essential functions, including synthesis and metabolism of carbohydrates, fats, and proteins, liver disease can potentially affect metabolism of both macro- and micronutrients. In canine chronic liver disease, several nutritional alterations are reported, and nutritional status can be affected. Many patients with liver disease can be underweight, with acute or chronic hyporexia, and it can be difficult to maintain the ideal body weight [37]. At the moment of inclusion 5 out of 16 (31.3%) patients presented inadequate MCS, whereas only one patient (6.3%) presented low BCS (<4/9). Sarcopenia is a possible syndrome in chronic liver disease, and these chronic patients may present muscular hypotrophy/atrophy as a consequence of their disease [38]. In our study, among CH dogs, MCS seemed to be more often inadequate if compared with BCS, which was normal in most CH dogs, despite the chronic disease. Further studies are needed to investigate a potential association of MCS with the AA alterations useful for the clinical evaluation, follow-up and prognosis in dogs with CH.

No difference in serum protein between the CH and healthy dogs was found (6.2 ± 1.1 vs. 6.4 ± 0.8, respectively). This result highlighted that even if proteinemia did not significantly differ between healthy and CH dogs, changes in the proportions of serum AAs may occur, reflecting qualitative amino-acidic imbalances. For this reason, clinicians should not underestimate these aspects in the management of chronic liver disease.

Hepatic fibrosis is often a sequela of canine chronic hepatitis. The development of fibrosis is a part of the progression of hepatic disease, which has shown prognostic value [39]. In advanced human cirrhotic patients, alteration in amino acids metabolism is well characterized. In those patients, serum concentrations of BCAAs (LEU, ILE, VAL) are decreased, whereas AAAs PHE and TYR, are increased, resulting in a decreased BCAAs/AAAs ratio, which is currently considered in the assessment of prognosis in cirrhotic human patients [8]. Our results were consistent with these findings since patients scored with a higher histological fibrosis grade (F3–4) showed a decreased BCAAs/AAAs ratio.

To the best of our knowledge, this is the first study attempting to associate histological fibrosis grade to serum AAs in canine CH. Before this study, reduced BCAAs/AAAs was found only in dogs with portosystemic shunts. We only observed reduced BCAAs/AAAs in dogs presenting advanced fibrosis degrees. Higher hepatic fibrosis during canine chronic hepatitis seemed to be associated with the same amino acid pattern observed in human patients. We should also consider that even if pathological mechanisms are similar, human and canine chronic hepatitis and cirrhosis may have different causes, thus every parallel should be considered with caution.

It was not possible to speculate on the pathogenetic mechanism of different aminoacidic trends observed in relation to both fibrosis and necroniflammatory activity scores, due to the scarce literature in veterinary medicine.

This study has several limitations. First at all, the low number of histological CH dogs. A larger group of dogs with chronic liver disease could better represent this complex pathological state. Considering the small number of cases, this study should be considered as a pilot-study. It is important to highlight that the non-standardized clinical conditions might affect the results. Moreover, although chronic hepatitis represents a specific pathological entity from a histological point of view regardless of the primary cause, we were not able to exclude possible implications of different etiologies on the amino acid pathway. The difference in mean age between the CH and C groups might also represent a bias in serum AAs concentration. Another limitation may be that the histological review of the bioptic specimens was undertaken by a single pathologist.

In the future, considering the importance of a comprehensive metabolomic approach, it is important to contextually evaluate other metabolites to better understand metabolic pathways. It would be useful to assess serum AAs during clinical follow-up in order to identify modifications along with the disease progression or improvement. Studies in humans have shown that supplementation of BCAAs improved nutritional status, prognosis and quality of life in patients with chronic liver disease [10,11,12,13]. BCAA and AAA imbalances may contribute to hepatic encephalopathy [40,41] and to decreased albumin synthesis [42,43,44].

One recent study in dogs with portosystemic shunts observed a reduction in PHE and TYR and a rise in VAL concentrations after surgery, showing the possible metabolic reversibility of these imbalances [19]. However, based on this study, we need further investigation to understand if some compounds may have a potential therapeutic role in the management of chronic liver disease. Deeper knowledge of serum AAs alteration in hepatopathic dogs may help to improve clinical and nutritional recommendations for support/restriction of these compounds in the management of those patients.

## 5. Conclusions

Serum AAs patterns differed significantly between CH dogs and clinically healthy dogs. Dogs diagnosed with chronic hepatitis and scored with higher histological fibrosis grades showed a decreased BCAAs/AAAs ratio. Even if proteinemia did not significantly differ, we observed changes in the proportions of serum AAs between healthy and CH dogs, reflecting qualitative AA imbalances.

## Figures and Tables

**Table 1:** Serum amino acids (nmol/mL) in group CH (T0) and in healthy group determined using HPLC technique, and expressed as mean ± SD or median and range. Statistical analysis: unpaired *t*-test (normally distributed data) or Mann–Whitney *u*-test (non-normally distributed data).

Amino Acid	Group CH	Group C	*p* Value
**GLY**	70.6 ± 21.8	48 ± 16.47	**0.0006**
**ALA**	78.7 (28.8–140.7)	94.5 (56.6–225.9)	0.06
**VAL**	34.9 (17.8–63.3)	4.8 (3–14.2)	**<0.0001**
**LEU**	26.7 (8.6–51.7)	10.7 (7.8–34.4)	**<0.0001**
**ILE**	9.3 ± 3.6	26.1 ± 8.3	**<0.0001**
**PRO**	55.9 (22.96–76.46)	6.3 (3.9–67.46)	**<0.0001**
**SER**	58.5 ± 23.6	41.3 ± 14.7	**0.006**
**THR**	45.4 (26.8–78.1)	50.2 (30.5–99.7)	0.2
**CYS**	5.8 ± 1.3	38.3 ± 15.3	**<0.0001**
**MET**	8.6 (3.4–36.6)	4.8 (0.3–58.5)	**0.003**
**PHE**	25.3 (10.4–63.1)	10.9 (4.5–24.6)	**<0.0001**
**TYR**	20.5 (11.42–67.72)	11.3 (6.3–54)	**0.0008**
**TRP**	19.8 (9.9–33.8)	48.2(19.7–108.3)	**<0.0001**
**ASP**	1.4 (0.14–5.36)	1.28 (0.16–5.46)	0.7
**GLU**	19.2 (4.9–102.6)	8.8 (4.4–20.6)	**0.001**
**HIS**	246.8 ± 82.7	198 ± 58.8	**0.03**
**LYS**	55.9 (29.1–96.4)	7.3(3.8–64.1)	**<0.0001**
**ARG**	54.2 (24.9–79.9)	59.2 (0.06–172.5)	0.6
**BCAAs/AAAs ratio**	1.89 (0.38–2.96)	1.72 (0.69–3.24)	0.9

**Table 2:** Histological scores A and F and respective number of assigned dogs.

Score	A Score (*n* = 16)	F Score (*n* = 16)
**0**	0	4
**1**	6	2
**2**	6	6
**3**	1	1
**4**	1	3
**5**	2	/
Total	16	16

## Data Availability

The complete data set is available upon reasonable request.

## Appendix A

**Table A1:** Descriptive statistics of serum hepatic enzymes (U/L), total protein (g/dL) and albumin (g/dL) expressed as medians and ranges in CH dogs at T0 time-point.

**Clinical Pathological Variable**	**Median (Range)**	**Reference Interval**
**ALP**	433 (122–2704)	45–250
**GGT**	7.3 (1.6–21.9)	2–11
**AST**	81.7 (24–565)	15–40
**ALT**	205 (64–987)	40–185
**Total serum proteins**	6.15 (4.4–8.5)	5.8–7.8
**Albumin**	3.2 (2.1–4.3)	2.6–4.1
